# The validation of a Japanese version of the New Freezing of Gait Questionnaire (NFOG-Q)

**DOI:** 10.1007/s10072-024-07405-y

**Published:** 2024-02-22

**Authors:** Seira Taniguchi, Kohei Marumoto, Yuta Kajiyama, Gajanan Revankar, Michiko Inoue, Hiroshi Yamamoto, Rika Kayano, Eiji Mizuta, Ryuichi Takahashi, Emi Shirahata, Chizu Saeki, Tatsuhiko Ozono, Yasuyoshi Kimura, Kensuke Ikenaka, Hideki Mochizuki

**Affiliations:** 1 Department of Neurology, Osaka University Graduate School of Medicine, 2-2 Yamadaoka, Suita, Osaka, 565-0871 Japan; 2 Hyogo Prefectural Rehabilitation Hospital at Nishi-Harima, 1-7-1 Koto, Shingu-Cho, Tatsuno, Hyogo Japan

**Keywords:** Freezing of gait, Parkinson’s disease, Measurement, Rating scale, Validation

## Abstract

**Objective:**

This study aimed to develop a Japanese version of the New Freezing of Gait Questionnaire (NFOG-Q) and investigate its validity and reliability.

**Methods:**

After translating the NFOG-Q according to a standardised protocol, 56 patients with Parkinson’s disease (PD) were administered it. Additionally, the MDS-UPDRS parts II and III, Hoehn and Yahr (H&Y) stage, and number of falls over 1 month were evaluated. Spearman’s correlation coefficients (rho) were used to determine construct validity, and Cronbach’s alpha (α) was used to examine reliability.

**Results:**

The interquartile range of the NFOG-Q scores was 10.0–25.3 (range 0–29). The NFOG-Q scores were strongly correlated with the MDS-UPDRS part II, items 2.12 (walking and balance), 2.13 (freezing), 3.11 (freezing of gait), and 3.12 (postural stability) and the postural instability and gait difficulty score (rho = 0.515–0.669), but only moderately related to the MDS-UPDRS item 3.10 (gait), number of falls, disease duration, H&Y stage, and time of the Timed Up-and-Go test (rho = 0.319–0.434). No significant correlations were observed between age and the time of the 10-m walk test. The internal consistency was excellent (α = 0.96).

**Conclusions:**

The Japanese version of the NFOG-Q is a valid and reliable tool for assessing the severity of freezing in patients with PD.

**Supplementary Information:**

The online version contains supplementary material available at 10.1007/s10072-024-07405-y.

## Introduction

Freezing of gait (FOG) is one of the most disturbing motor symptoms in Parkinson’s disease (PD) [1], affecting 60%–80% of patients with PD [2]. This increases the risk of falls [3] and reduces the quality of life [4]. Despite its common and disabling features in everyday circumstances, it is challenging to assess the severity of FOG in daily clinical practice because of its episodic nature [5].

The New Freezing of Gait Questionnaire (NFOG-Q) has been widely used to subjectively quantify the occurrence and severity of FOG [6, 7]. The NFOG-Q is the only recommended instrument for assessing FOG severity [8, 9], as it has adequate clinimetric properties and the benefits of being short and easy to administer [7, 8].

The NFOG-Q consists of nine items organised into three parts: “Part I – Distinction (freezer or non-freezer)”, “Part II – Freezing severity”, and “Part III – Freezing impact on daily life”, which together assess FOG severity and its impact on daily life. Another feature of the NFOG-Q is a video demonstration of the FOG for participants to view different freezing episodes before completing the questionnaire, which allows them to accurately perceive the FOG. This leads to more reliable outcomes of the questionnaire-based methodology [7].

To date, the original version of the NFOG-Q has been translated into several languages, including Swedish [10], Italian [11], Turkish [12], Spanish [13], Brazilian Portuguese [14], Czech [15], German [16], Chinese [17], and Thai [18] (in order of publication year).

Despite their advantages and global use in assessing FOG severity, a Japanese translation is not currently available. Therefore, the aim of the current study was to develop a Japanese version of the NFOG-Q and investigate its validity and reliability.

Furthermore, the pathophysiological mechanism of FOG has been implicated in executive dysfunction as a series of functional neuroimaging studies have highlighted that frontostriatal projections and the basal ganglia hyperdirect pathway may contribute to FOG [19–21]. Thus, we further investigated the relationship between the NFOG-Q and executive function as well as global cognition.

## Methods

### Participants

Sixty-one patients with PD from two different medical centres (*N* = 32, Osaka University Hospital, Japan; *N* = 29, Hyogo Prefectural Rehabilitation Hospital at Nishi-Harima, Japan) participated in the study. The inclusion criteria were as follows: (1) diagnosis of idiopathic PD based on the Movement Disorder Society (MDS) clinical diagnostic criteria [22] and (2) Hoehn and Yahr (H&Y) stage < 5. The exclusion criteria were as follows: (1) probable dementia (Mini-Mental State Examination (MMSE) score < 24) [23] and (2) problems other than PD that might affect gait.

### Development of the Japanese version of the NFOG-Q

After obtaining permission from the developers of the original version [6, 7], the Japanese version of the NFOG-Q was developed in accordance with a standardised protocol [24] (see Supplementary Information).

### Procedure

Demographics and disease profiles, including the H&Y stage (“on,” or best state), MDS-sponsored revision of the Unified Parkinson’s Disease Rating Scale (MDS-UPDRS) parts II and III, medication (Levodopa Equivalent Daily Dose [LEDD]), number of falls over the previous 1 month, and cognitions (Mini-Mental State Examination [MMSE], and the Frontal Assessment Battery [FAB]) were evaluated. Additionally, participants underwent a clinical evaluation using the 10-m walk test (10MWT) and the Timed Up-and-Go test (TUG).

After participants watched the demonstration video, they were administered the NFOG-Q. The questionnaire’s nine items have ratings of up to 3 or 4, with a total score ranging from 0 to 29 points, with a higher score indicating more severe FOG.

Construct validity was examined by determining the associations between the NFOG-Q scores and functional measures (scores on the MDS-UPDRS, number of falls, and objective gait measures). Score reliability and ceiling and floor effects were also evaluated based on previous validation studies [6, 11]. Participants were classified as “faller” if they had at least one fall, or “non-faller” if they had no history of fall within the last 1 month. Moreover, participants were categorized as the “freezers” if they had a score of > 0, or “non-freezers” if they had a score of = 0 on the NFOG-Q, and we investigated the relationship between the NFOG-Q and global cognition (measured by the MMSE) and executive function (measured by the FAB) in whole group as well as the freezers. The questionnaires and other assessments were completed during the participants’ ‘‘*on*’’ or best state. This study was conducted after pre-registration with the UMIN Clinical Trials Registry (UMIN000049889). All participants provided informed consent as required under the Declaration of Helsinki. The study protocol was approved by the Ethics Committee of Osaka University Hospital (No. 20563) and the Hyogo Prefectural Rehabilitation Hospital at Nishi-Harima (No. 2209). Data collection procedures were harmonized and standardized across centres.

### Statistical analysis

Construct validity of the NFOG-Q was determined based on correlations between the NFOG-Q scores and existing functional scales: the MDS-UPDRS items 2.12 (Walking and balance), 2.13 (Freezing), 3.10 (Gait), 3.11 (Freezing of gait), 3.12 (Postural Stability); the MDS-UPDRS part II total; MDS-UPDRS part III total; postural instability and gait difficulty (PIGD) score, which is the sum of sub-items 2.12, 2.13, 3.10, 3.11, 3.12 [25]; TUG time, fast gait time; and number of falls. Spearman’s correlation coefficients (rho) were considered moderate for values of 0.30–0.39 and strong for values of 0.40–0.69 [26].

To determine reliability, the internal consistency was examined using Cronbach’s alpha (α) for which a value > 0.90 was considered excellent internal consistency [27].

To evaluate the possible ceiling or floor effects, the proportion of participants obtaining the minimum and maximum scores was set at a threshold of 15% [28]. Additionally, a receiver operating characteristic (ROC) curve analysis was conducted to examine the optimal NFOG-Q cutoff value for distinguishing fallers from non-fallers.

Statistical analyses were performed using SPSS (version 29.0). The false discovery rate (FDR)-corrected *P* values < 0.05 were considered statistically significant.

## Results

### Patient characteristics and NFOG-Q score distributions

Fifty-six patients were included in this study after excluding five patients. The median age was 71.0 years (interquartile range [IQR] 65.5–76.0), with a median disease duration of 11 years (IQR 6.8–14.0); a median UPDRS-III score of 30.5 (IQR 19.75–42.25); and H&Y stage II (*n* = 11), III (= 35), and IV (*n* = 10). The median total NFOG-Q score was 21.0 (IQR 10.0–25.3) with a range of 0–29, and 48 patients were classified as freezers and 8 patients as non-freezers. The floor and ceiling effects were both < 15%. Twenty-two participants (39.3%) had experienced at least one fall during the previous 1 month. Further characteristics of the participants are presented in Table 1.

**Table 1:** Participant demographics, *n* = 56 (20 women, 36 men)

	Median	IQR q1–q3 (min–max)
NFOG-Q score (range 0–29)	21.0	10.0–25.3 (0–29)
Age (yr)	71.0	65.5– 76.0 (53–86)
Disease Duration (yr)	11.0	6.8–14.0 (2–40)
Hoehn and Yahr stage (2/3/4)	*n* = 11/25/10	
Levodopa equivalent daily dose (mg)	600.0	436.9–900.0 (25–1650)
MDS-UPDRS part II total (range 0–52)	13.5	9.0–22.0 (2–31)
MDS-UPDRS item 2.12, Walking and balance (range 0–4)	2.0	1.0–3.0 (0–4)
MDS-UPDRS item 2.13, Freezing (range 0–4)	2.0	1.0–3.0 (0–4)
MDS-UPDRS part III total (range 0–132)	30.5	19.8–42.3 (8–73)
MDS-UPDRS item 3.10, Gait (range 0–4)	1.5	1.0–3.0 (0–4)
MDS-UPDRS item 3.11, Freezing of gait (range 0–4)	1.5	1.0–3.0 (0–4)
MDS-UPDRS item 3.12, Postural Stability (range 0–4)	2.0	1.0–3.0 (0–4)
MDS-UPDRS PIGD score (range 0–20)	10.0	5.0–14.0 (0–20)
Mini Mental State Examination (range 0–30)	28.0	26.0–29.0 (24–30)
Frontal assessment battery (range 0–18)	14.5	13.0–17.0 (8–18)

IQR: Interquartile range

q1: The first quartile indicates the 25th percentile

q3: The third quartile indicates the 75th percentile

NFOG-Q: a Japanese version of the New Freezing of Gait Questionnaire

MDS-UPDRS: MDS-sponsored revision of the Unified Parkinson’s Disease Rating Scale

PIGD: Postural instability and gait difficulty, sum of sub-items 2.12, 2.13, 3.10, 3.11, 3.12

### Validity and reliability

#### Construct validity

Correlation analysis showed that the NFOG-Q score was significantly positively related to the MDS-UPDRS items 2.12 (walking and balance), 2.13 (freezing), 3.11 (freezing of gait), and 3.12 (postural Stability), PIGD score, and total scores in the MDS-UPDRS parts II and III and positively correlated with disease duration, H&Y stage, TUG time, and number of falls. However, there was no significant association between the NFOG-Q score and age or fast gait time during the 10MWT. Additional information is presented in Table 2.

**Table 2:** Correlations with the NFOG-Q score (*n* = 56)

	Correlation (rho)	*p*-value
Age (yr)	0.037	0.787
Disease Duration (yr)	**0.390**	0.005^**^
Hoehn and Yahr stage	**0.394**	0.005^**^
MDS-UPDRS part II total	**0.599**	0.000^**^
MDS-UPDRS item 2.12, Walking and balance	**0.599**	0.000^**^
MDS-UPDRS item 2.13, Freezing	**0.669**	0.000^**^
MDS-UPDRS part III total	**0.353**	0.010^**^
MDS-UPDRS item 3.10, Gait	**0.374**	0.007^**^
MDS-UPDRS item 3.11, Freezing of gait	**0.515**	0.000^**^
MDS-UPDRS item 3.12, Postural Stability	**0.662**	0.000^**^
MDS-UPDRS PIGD score	**0.663**	0.000^**^
TUG time (s; *n* = 54)^a^	**0.319**	0.022^*^
Fast gait time (s; *n* = 54)^b^	0.109	0.466
Number of falls (m)	**0.434**	0.002^**^

Significant results are shown in bold font. * *p* < 0.05, ** *p* < 0.01, the false discovery rate (FDR)-corrected

PIGD: Postural instability and gait difficulty, sum of sub-items 2.12, 2.13, 3.10, 3.11, 3.12

^a^Timed Up and Go Test

^b^10-meter walk test

#### Predictive validity 

ROC analysis of the NFOG-Q score indicated an optimal cut-off value of 20.5 points for distinguishing fallers from non-fallers over 1 month, with sensitivity of 81.8% and specificity of 61.8%, and the area under the ROC curve (AUC) was 0.72.

#### Reliability 

Internal consistency as measured by Cronbach α was 0.96, suggesting adequate reliability.

### Association between scores in the NFOG-Q and cognitive performances

There were no significant correlations between scores in the NFOG-Q and cognitive tests in any freezer group (with the MMSE [rho = -0.112, *p* = 0.689], and with the FAB [rho = 0.059, *p* = 0.689]), or whole PD group (with the MMSE [rho = -0.092, *p* = 0.497], and with the FAB [rho = 0.266, *p* = 0.096]).

## Discussion

A Japanese version of the NFOG-Q was developed and its validity and reliability were evaluated. The construct validity of the NFOG-Q was demonstrated by a good correlation with freezing- and gait-related measures and negligible floor and ceiling effects. The Japanese version of the NFOG-Q also showed high internal consistency (α = 0.96), which was similar to the results of original English version (0.96) [6] and other validation studies (0.81–0.95) [10, 11, 13–15, 17].

Correlation analysis showed that the NFOG-Q score was related to the TUG test time; however, it was not related to fast gait time during the 10MWT. This could be explained by the higher complexity of the TUG test (incorporating chair transfer, walking, and turning) compared with the straight-path walking in the 10MWT [29], and there is a consensus that turning is one of the strongest provocative triggers of FOG [30–33].

Consistent with previous translated versions [6, 10, 14, 15, 17], the current results demonstrate the relationship between the NFOG-Q score and the number of falls over one month. Unsurprisingly, 61% of falls in PD are related to FOG (12.6% syncope, 26.3% imbalance) [3]. The current study showed that the NFOG-Q scores had a stronger relationship with freezing items than with gait items: item 2.13 (freezing) vs item 2.12 (walking and balance), rho = 0.669 vs 0.599; item 3.11 (freezing of gait) vs item 3.10 (gait), rho = 0.515 vs 0.374, respectively.

Moreover, our findings indicated that subjectively reported items (i.e., MDS-UPDRS part II total and items 2.12 and 2.13) were more strongly related to NFOG-Q scores than objectively measured items of the MDS-UPDRS (i.e., MDS-UPDRS part III total and items 3.10 and 3.11), similar to previous translations [10, 11, 15, 17, 18]. To explain these results, the effect of different types of measurements (subjective or objective) on the freezing phenomenon should be considered. Importantly, it is recognised that gait can improve and freezing of gait can disappear while an examiner observes [5, 34], since patients shift from an automatic motor control to a more goal-directed one [35], which is also described as the "white coat effect" [34]. In particular, the current study enrolled only inpatients and outpatients who were assessed in a hospital setting, which may have resulted in a better performance in hospitals than in daily life settings.

Moreover, we found that the NFOG-Q score was related to disease duration and H&Y stage but not to age, consistent with other validation studies [10, 11, 14, 18]; however, one validation study showed that the NFOG-Q was associated with increased age [15]. This discrepancy may be ascribed to the differences in the study populations, given that the four studies reporting no correlation with age enrolled a relatively large number of participants compared with that reporting a correlation [10, 11, 14, 18]. In addition, a large-cohort survey (6,620 patients) further supported this interpretation, showing that freezing episodes were significantly related to a longer disease duration and a more advanced stage of PD but had no relationship with age [36].

Furthermore, the fall rate in this study was similar to that of the Chinese validation study (39.3% vs. 32.4%) [17] and the results of the ROC analysis indicated good predictive validity. The cut-off value for the NFOG-Q was 20.5 points for distinguishing fallers from non-fallers over 1 month, while a previous study showed a cut-off of 5.5 points over a 12-month period [37]. Thus, a follow-up study is required to examine prospective fall histories over long intervals.

We did not find any significant associations between the scores in the cognitive functions and the NFOG-Q. They are in keeping with a recent novel study including a large cohort of PD, which showed that the NFOG-Q scores was not associated to global cognition or executive function [38]. However, more in depth assessment of more specific executive function (such as the behavioral assessment of the dysexecutive syndrome battery [39]) may be needed to rule out the dependency of the cognitive functions.

One limitation of the current study was that non-motor symptoms (e.g., anxiety, stress, depression), which could also affect FOG [40] were not adequately assessed, as only the MMSE and the FAB considered cognitive impairments in this study. However, further studies are required to clarify this.

In conclusion, the current study developed a Japanese version of the NFOG-Q and reported its validity and reliability in assessing FOG in patients with PD.

### Supplementary Information

Below is the link to the electronic supplementary material.

Supplementary file1 (MP4 80668 KB)

Supplementary file2 (DOC 54 KB)

## Data Availability

The datasets generated and analysed during the current study are not publicly available because of ethical restrictions but are available from the corresponding author on reasonable request.
