# Supplementary material for: The validation of a Japanese version of the New Freezing of Gait Questionnaire (NFOG-Q)
Source: Neurol Sci. 2024 Feb 22;45(7):3147–52. doi: 10.1007/s10072-024-07405-y (PMC11176215; doi:10.1007/s10072-024-07405-y)
Supplement: Supplementary file 2 — Supplementary file2 (DOC 54 KB) [file 10072_2024_7405_MOESM2_ESM.doc]

日本語版NFOG-Q (New Freezing of Gait Questionnaire)

| パートⅠ - 　過去1ヵ月に足がすくんだ経験の有無 |
| --- |
| 1. 過去1ヵ月に「すくみ足」を経験しましたか？   *ビデオなし*  *すくみ足とは、歩き始めようとした時、方向転換をしようとする時、または狭い場所や人混みの中を通ろうとした時などに、一時的に足が地面にくっついているように感じることです。足の震えや引きずりを伴うこともあります。*  *ビデオがある場合の追加説明*  *すくみ足が起こりうるいろいろな場面について、一緒にショートビデオを見てみましょう。また、このような状態がどのくらい続くのか、注意して見てください。持続時間について、この後にお尋ねすることがあります。　　（係の者がビデオクリップ上の時計を指します）*  ０　過去1ヵ月に、このような感じや状態を経験したことはない。  １　過去1ヵ月に、このような感じや状態を経験した。  *回答が1であれば（すくみ足患者）、パートⅡとⅢに進みます。*  *パートⅡとⅢの合計点が、最終的なNFOGスコアとなります。* |
| パートⅡ – 　すくみ足の重症度 |
| 2． どのくらいの頻度ですくみ足が生じていますか？   1. 週に1回もない 2. それほど多くなく、週に1回程度  しばしば生じ、1日に1回程度  1. 非常に多く、1日に2回以上 |
| 3． 方向転換をしている間に、どのくらいの頻度で足がすくみますか？   1. 全くなし 2. たまにすくむが、1ヵ月に1回程度 3. それほど多くはなく、週に1回程度 4. しばしばすくみ、1日に1回程度 5. 非常に多く、1日に2回以上   *回答が1以上（1,2,3,4）であれば、質問4に進みます。*  *回答が0であれば、質問4を飛ばして質問5に進みます。* |
| 4． 方向転換をしている間に足がすくんだ時、最も長かった持続時間は  　　 どのくらいですか？   - - - 1. 非常に短く、1秒程度       2. 短く、2～5秒程度       3. 長く、5～30秒程度       4. 非常に長く、30秒以上歩けなかった |
| 5． 最初の一歩を踏み出そうとした時、どのくらいの頻度で足がすくみますか？   1. 全くなし 2. たまにすくむが、1ヵ月に1回程度 3. それほど多くはなく、週に1回程度 4. しばしばすくみ、1日に1回程度 5. 非常に多く、1日に2回以上   *回答が1～4であれば、質問6に進みます。*  *回答が0であれば、質問6を飛ばして質問7に進みます。* |
| 6． 最初の一歩を踏み出そうとして足がすくんだ時、最も長かった持続時間はどのくらいですか？   1. 非常に短く、1秒程度 2. 短く、2～5秒程度 3. 長く、5～30秒程度 4. 非常に長く、30秒以上歩けなかった |
| **パートⅢ – すくみ足が日常生活に及ぼす影響** |
| - 1. 毎日の歩行で、すくみ足がどの程度問題となっていますか？  1. 全く問題なし 2. ほとんど問題なし 3. ある程度問題あり 4. 非常に問題あり |
| - 1. 足がすくむことで、不安が生じたり、転倒の恐れを感じたりしますか？  1. 全く感じない 2. ほとんど感じない 3. ある程度感じる 4. 非常に感じる |
| 9．すくみ足は日常の活動に影響を及ぼしていますか？  （病気の全般的な影響ではなく、日常の活動にすくみ足が及ぼす影響のみを  評価してください）   1. 全く影響なし。いつも通り活動を続けている。 2. ほとんど影響なし。制限される活動はわずかしかない。 3. 影響あり。かなり多くの活動（ほぼ半分）が制限されている。 4. 非常に影響あり。ほとんどの活動が制限されている。 |
